# Fantastic voyage: the journey of intestinal microbiota-derived microvesicles through the body

**DOI:** 10.1042/BST20180114

**Published:** 2018-08-28

**Authors:** Régis Stentz, Ana L. Carvalho, Emily J. Jones, Simon R. Carding

**Affiliations:** 1 Gut Microbes and Health Programme, Quadram Institute Bioscience, Norwich Research Park, Norwich NR4 7UA, U.K.; 2 Norwich Medical School, University of East Anglia, Norwich, U.K.

**Keywords:** *Bacteroides*, gut microbiota, host–microbe interactions, outer membrane vesicles, vaccine

## Abstract

As part of their life cycle, Gram-negative bacteria produce and release microvesicles (outer membrane vesicles, OMVs) consisting of spherical protrusions of the outer membrane that encapsulate periplasmic contents. OMVs produced by commensal bacteria in the gastrointestinal (GI) tract of animals are dispersed within the gut lumen with their cargo and enzymes being distributed across and throughout the GI tract. Their ultimate destination and fate is unclear although they can interact with and cross the intestinal epithelium using different entry pathways and access underlying immune cells in the lamina propria. OMVs have also been found in the bloodstream from which they can access various tissues and possibly the brain. The nanosize and non-replicative status of OMVs together with their resistance to enzyme degradation and low pH, alongside their ability to interact with the host, make them ideal candidates for delivering biologics to mucosal sites, such as the GI and the respiratory tract. In this mini-review, we discuss the fate of OMVs produced in the GI tract of animals with a focus on vesicles released by *Bacteroides* species and the use of OMVs as vaccine delivery vehicles and other potential applications.

## Introduction

The production and release of membrane vesicles from microbial surfaces is a process that is conserved across the three domains of life. This includes Gram-negative and Gram-positive bacteria, archaea, fungi and protozoa [1]. It is only recently that compelling evidence for the production of EVs by microorganisms with thick cell walls, such as Gram-positive bacteria, mycobacteria and fungi, has been obtained [2]. Here, we focus on the more advanced findings of extracellular vesicles produced by Gram-negative bacteria and, in particular, by representatives of the genus *Bacteroides* that are prominent members of the vertebrate intestinal microbiota.

Gram-negative bacteria produce outer membrane vesicles (OMVs) which are spherical buds of the outer membrane that encapsulate periplasmic components. The molecular mechanism of their biogenesis is still unclear, although several models of OMV formation have been proposed and have been recently reviewed [3,4]. OMVs range in size from 20 to 400 nm and can transport a variety of biomolecules such as enzymes, toxins, antigenic determinants, nucleic acids [5] and metabolites [6,7]. OMV contents are protected from enzymatic degradation by a lipid bilayer envelope that protects against the harsh extracellular environments of the GI tract [8,9]. Here, we review our current understanding of the fate of OMVs produced in the GI tract of animals with a particular focus on those released by commensal *Bacteroides* species. We also provide a brief update on the use of OMVs as mucosal delivery vehicles for biologics.

## The fate of OMVs produced by commensal bacteria in the GI tract of animals

### OMVs and digestion

**Figure 1.**
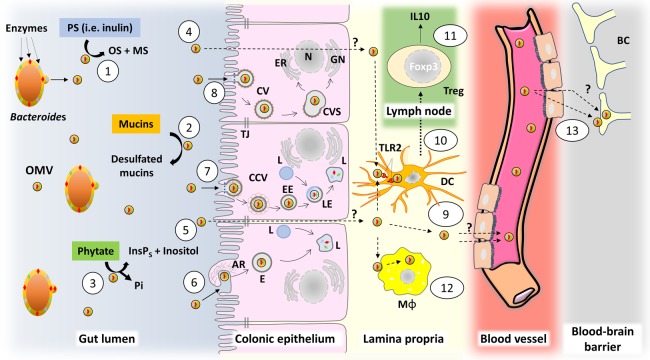
The predicted journey of a bacterial membrane vesicle from the gut to the brain. The schematic depicts the (numbered) pathways and means by which OMVs produced in the lower GI tract by members of the intestinal microbiota can access and cross the epithelial barrier to gain access to underlying immune cells and the systemic circulation to access other organ systems and possibly the brain. 1. Degradation of polysaccharides by OMVs. 2. Mucin sulfatase activity carried by OMVs. 3. Degradation of inositol polyphosphates (i.e. phytate) by OMVs. 4. Transcellular transmigration. 5. Paracellular transmigration. 6. Macropinocytosis. 7. Clathrin-mediated endocytosis. 8. Caveolin-mediated endocytosis. 9. TLR2-dependent OMV internalisation by DCs. 10. DC migration. 11. Induction of Treg by DC modulated by OMVs. 12. Internalisation of OMVs by macrophages. 13. Hypotheses of OMV translocation across the blood–brain barrier. Abbreviations: OMV, outer membrane vesicle; PS, polysaccharide; OS, oligosaccharide; MS, monosaccharide; InsPs, inositol polyphosphates; TJ, tight junction; AR, actin remodelling; ER, endoplasmic reticulum; N, nucleus; GN, Golgi network; E, endosome; L, lysosome; CCV, clathrin-coated vesicle; EE, early endosome; LE, late endosome; CV, caveolar vesicle; CS, caveosome; DC, dendritic cell; Treg, regulatory T cell; Mφ, macrophage; BC, brain cells.

OMVs from Gram-negative bacterial species are produced and released into the intestinal lumen [10,11] enabling them to affect their environment remotely from their parent cells. Members of the *Bacteroides* genus distribute hydrolases including proteases and glycosidases [12] within the GI tract lumen using OMVs as delivery vehicles (Figure 1) that contribute to the communal breakdown of complex polysaccharides, the products of which serve as a source of nutrients for other members of the intestinal microbiota and the host [13]. Other examples of enzymes distributed via *Bacteroides* OMVs include multiple inositol polyphosphatases [8] which degrade dietary phytate to release phosphate, inositol phosphates and inositol (Figure 1), mucin sulfatases [10] that make mucin glycans more susceptible to degradation by bacterial glycosidases (Figure 1) and β-lactamases involved in antibiotic resistance which contributes to antibiotic resistance of other members of the microbiome [14].

### Interaction with the intestinal mucosa

#### OMV–epithelial cell interactions

Gram-negative OMVs can use several routes to cross the intestinal epithelial barrier, which differ according to bacterial species [15]. Both phagocytic and non-phagocytic pathways have been implicated in host–cell interactions with the GI tract, although it should be noted that most studies have, to date, focused on OMVs of pathogenic rather than commensal bacteria. The observation that microbiota-derived OMVs are phagocytosed by immune cells in the lamina propria [16] and can be detected in the blood and urine [17,18] suggests that OMVs can cross the intestinal epithelium and vascular endothelium to reach sites beyond the GI tract. To do this, OMVs can utilise two distinct pathways to cross the intestinal epithelium or vascular endothelium: the paracellular (between cells) or transcellular (through cells) pathways.

Pathogenic OMVs can alter the intestinal barrier by modulating the permeability of cellular junctional complexes. *Campylobacter jejuni* OMVs cleave the adherens junction proteins E-cadherin and the tight junction (TJ) protein occludin [19]. *Helicobacter pylori* OMVs, containing the cytotoxin CagA, localise in the vicinity of cellular junctions and cause the redistribution of the TJ protein ZO-1 to the cytoplasm [20]. Among the few studies to date on commensal OMV–host cell interactions, the probiotic *Escherichia coli* Nissle 1917 strain has been shown to up-regulate expression of barrier-enhancing ZO-1 and ZO-2 proteins [21]. Together, these studies suggest that OMVs can directly or indirectly alter the composition of junctional complexes to enable them to cross epithelial barriers with this mechanism of trafficking being associated with virulence. It is possible that OMVs produced by commensal bacteria may also reduce TJ permeability, potentially limiting paracellular transport [22]. A challenge to identifying the specific routes of uptake and trafficking through host cells is visualising the localisation of nanosize OMVs and OMV-cargo alongside host proteins and organelles.

Using chemical inhibitors of endocytic pathways, OMVs have been shown to use all four endocytosis pathways: actin-dependant macropinocytosis, clathrin-mediated endocytosis, caveolin-mediated endocytosis or clathrin- and caveolin-independent mechanisms such as membrane fusion or lipid raft formation (reviewed in ref. [5]). Identifying the specific pathways of OMV and OMV-associated cargo entry into host cells has, however, proved challenging as chemical inhibitors are not always specific [23]. Also, as OMV preparations are heterogenous and consist of a range of vesicle sizes, they are likely to be internalised in a size-dependant manner, with potentially several endocytic pathways in simultaneous use [24–27].

The majority of endocytic routes of OMV uptake culminate in sequestration and degradation by lysosomes. Depending on the mechanism of endocytosis, internalised OMVs generally join either the host endolysosomal pathway or autophagy pathway (Figure 1). Caveolin-dependant endocytosis is an exception as it involves invagination of the host plasma membrane and delivery into specialised endosomes termed caveosomes which transport their cargo via the cytoskeleton to the host endoplasmic reticulum/Golgi complex. As this route of uptake does not merge with lysosomes, it may be utilised by OMVs to escape lysosomal degradation (Figure 1).

#### OMV–immune cell interactions

There are several lines of evidence that OMVs produced by *Bacteroides* interact with the mucosal immune system. Shen et al. [16] have demonstrated that OMVs from *Bacteroides fragilis* harbouring the capsular polysaccharide A (PSA) are sensed and internalised by dendritic cells (DCs) in an actin-dependent manner involving the Toll-like receptor (TLR) 2. This results in enhanced regulatory T cells (Treg) and production of the anti-inflammatory cytokine IL-10 as well as the Foxp3 transcription factor (Figure 1), a marker of Treg cells associated with protection from inflammatory and autoimmune diseases [28,29]. This finding has been interpreted as OMVs inducing host immunological tolerance to PSA or OMV-associated PSA. Within the context of OMVs promoting a protective effect in experimental models of colitis, Chu et al. [30] have shown that OMVs require the Crohn's disease (CD) susceptibility alleles ATG16L1 and NOD2, to activate a non-canonical autophagy pathway. For example, ATG16L1-deficient DCs lose the ability to induce Treg which in turn compromises the suppression of mucosal inflammation. Moreover, immune cells from human subjects with a major CD risk variant in the ATG16L1 allele are defective in Treg responses to OMVs [30]. Hickey et al. [10] have shown that access of *Bacteroides thetaiotaomicron* OMVs to the mucosal immune system is dependent on the activity of one or more sulfatases. Considering that mucin glycans are protected from enzymatic breakdown by sulfate residues [31,32], it is tempting to speculate that localised mucin degradation by OMVs facilitates their access to the intestinal epithelium and underlying cells of the mucosal immune system.

### OMVs cross the intestinal mucosa and enter the bloodstream

#### Detection of OMVs in the blood

Results from various studies suggest that serum from healthy individuals contains genomic DNA of bacterial origin [33–35] in a phenomenon termed DNAemia. In these studies, DNA was extracted from blood samples using protocols primarily designed for bacterial cell disruption and cell lysis during denaturation steps in PCRs. While the presence of these blood-associated bacterial DNA sequences was inexplicable when first described [33], it has recently been shown in a mouse model that blood-linked bacterial DNA is associated with bacterial membrane microvesicles that appear to be natural blood compounds, the diversity of which reflects the bacterial diversity of the intestinal microbiota [17]. This finding implies that bacterial microvesicles produced by bacteria in the GI tract may normally cross the intestinal epithelial barrier to reach the bloodstream. In their study, Park et al. claimed that based upon metagenomic sequencing, blood-derived microvesicles represent an alternative to faecal sampling for profiling the human intestinal microbiota and could constitute a new tool for evaluating variations of the intestinal microbiota (microbial dysbiosis) in the context of neurodegenerative diseases.

#### Nucleic acids of bacterial origin in the brain

Access to the brain via the blood stream is controlled primarily by the blood–brain barrier (BBB) composed of brain microvascular endothelial cells which line brain capillaries and effectively regulate molecular and cellular trafficking between the bloodstream and neural tissue. Maintaining the integrity of the BBB is essential for limiting the entry of potentially neurotoxic plasma components, blood cells and pathogens into the brain [36,37]. Additionally, TJs and adjacent perivascular cells, including astrocytes and pericytes, reinforce the BBB function [38]. A handful of studies have provided evidence for the presence of rRNA and rDNA from commensal bacteria in the brain and that it may have a unique microbiota dominated by fastidious obligate intracellular α-proteobacteria [39–41]. Although one study [39] has provided evidence of culturable bacteria in the brain, the majority of studies have relied on culture-independent, sequence-based approaches to identify brain-associated microbes. In view of the presence of bacterial microvesicles in the bloodstream and their ability to cross boundary epithelial cells, it is interesting to speculate that a fraction of theses microbial nucleic acids might originate from, and gain access to, the brain via bacterial microvesicles. Alternatively, microvesicles may also be produced by brain-resident bacteria.

## OMV-based vaccines

The nanosize and non-replicative status of OMVs together with their resistance to enzymes and low pH, alongside their ability to interact with mucosal and systemic host cells, makes them ideal candidates for drug delivery. Moreover, they possess innate adjuvant properties and the ability to activate immune cells via interactions between microbe-associated molecular patterns (MAMPs) or capsular polysaccharides they carry and TLRs on the surface of host antigen-presenting cells and, in particular, DCs [16].

### Meningococcal OMV vaccines

Commercial formulations of *Neisseria meningitidis*-generated OMV vaccines have been used for epidemic control of group B *N. meningitis* infections in New Zealand and Cuba [42,43]. In these outbreaks, group B *N. meningitis* OMVs were effective in controlling infection and conferring protection in both adults and children [42,43]. More recently, a meningitis serogroup B vaccine, Bexsero, which contains *N. meningitis* OMVs, was approved by the European Medicines Agency (EMA) and the Food and Drug Administration (FDA) [44].

The *N. meningitis* OMV vaccines used, to date, in commercial preparations are administered parenterally [45] although several reports have shown the effectiveness of meningococcus OMV vaccines at eliciting mucosal responses [46–48]. OMVs from other Gram-negative bacteria have been formulated for use as experimental vaccines, with some showing induction of protective immune response upon mucosal administration via the intranasal or oral route [49–51].

### Development of new mucosal OMV vaccines

The high stability of OMVs together with their ability to interact with the host immune system makes them attractive candidates for the development of new mucosal vaccines. As mucosal sites are the main location of entry of most pathogens, with induction of the mucosal immune system and neutralising (IgA) antibody production being required for immune protection, the development of such vaccines is of extreme importance, especially in light of the increase in antibiotic resistance among major bacterial pathogens.

Studies carried out to date using pathogen-derived and modified OMV vaccine formulations show that they are effective at inducing protective systemic and mucosal antigen-specific antibody and T-cell responses. Of interest, Schager et al. [52] have recently shown that administration of OMVs produced by hyperblebbing strains of *Salmonella* Typhimurium can generate protective antibodies in mice. OMVs produced by different bacteria are equipped with specific sets of proteins that can bind to receptors on host intestinal antigen sampling M cells and DCs. M cells are present in the follicle-associated epithelium of the small intestine as well as the colon and rectum and are responsible for the sampling and uptake of luminal particulate antigens. Luminal antigens can also be acquired by DCs either directly or indirectly via epithelial cells that then initiate immune responses via activation of antigen-specific B cells and T cells.

M cells express an array of receptors that enable them to recognise various food/microbe/host cell-derived ligands that have been exploited to enhance M cell uptake by mucosal vaccines. For example, nanoparticles coated with UEA-1, a specific lectin and ligand for α-l-fucose present on the apical membrane of M cells, are able to effectively induce both mucosal and systemic immune responses in mice [53]. Bacterial OMVs, which have been used as vaccines (described above), are decorated with MAMPs which allow them to interact with the immune system, in particular, they can be recognised by TLR-4 and α5β1 integrin receptors present in M cells [54,55]. M cell-restricted receptors have been identified including PrPC and the C5a molecules, which interact with the Hsp60 of *Brucella abortus* and with OmpH of *Yersinia enterocolitica*, respectively [56,57]. Another example is glycoprotein 2 (GP2) expressed on the luminal surface of M cells which interacts with the type-I pili of bacterial outer membranes [58]. Type-I pili are composed of FimA monomers and are present in Gram-negative pathogens including *E. coli* and *Salmonella* Typhimurium [59]. The genes encoding FimA superfamily proteins are often represented in the intestinal microbiome and in particular, in *Bacteroides* genomes where they can be found as putative pilus biogenesis operons [60]. Our proteomic analysis of OMVs produced by *B. thetaiotaomicron* has revealed at least one FimA-like protein (unpublished data). Thus, OMVs from bacteria containing FimA-like proteins, Hsp60 or OmpH might be ideal candidates for mucosal antigen delivery vaccines.

## Concluding remarks

Extracellular microbial structures, such as OMVs and more generally microvesicles, are viewed as a new type of secretion system that enables the dissemination of membrane-encapsulated cellular materials including proteins, nucleic acids and metabolites into the extracellular milieu [4]. Microvesicles released by commensal microorganisms into the intestinal lumen have the potential to contribute to host physiology including digestion by distributing hydrolase activities across the lumen to help maximise digestion of macromolecules. They are internalised by boundary epithelial cells using different routes. They can also cross epithelial barriers enabling them to interact with mucosal immune cells to induce host immunological tolerance and to disseminate more widely around the body and perhaps the brain, via the bloodstream. It is becoming clear that microvesicles produced by commensal bacteria can mediate interkingdom cross-talk between the intestinal microbiota and the host and that these interactions confer a health benefit to the host. In addition, the potential of OMV as vehicles for the delivery of biologics and vaccine antigens is beginning to emerge. Different approaches to expressing heterologous proteins in these microvesicles [61,62] open new possibilities for the use of OMVs as vaccine delivery vehicles with significant advantages over current vaccine strategies and those that aim to protect mucosal surfaces from pathogen invasion.

## Abbreviations

BBB: blood–brain barrier
CD: Crohn's disease
DC: dendritic cell
GI: gastrointestinal
MAMPs: microbe-associated molecular patterns
OMV: outer membrane vesicle
PSA: polysaccharide A
TJ: tight junction
TLR: Toll-like receptor
Treg: regulatory T cell

## References

[1] Deatherage B.L. and Cookson B.T. (2012) Membrane vesicle release in bacteria, eukaryotes, and archaea: a conserved yet underappreciated aspect of microbial life. Infect. Immun. 80, 1948–1957 10.1128/IAI.06014-11 22409932 PMC3370574

[2] Brown L., Wolf J.M., Prados-Rosales R. and Casadevall A. (2015) Through the wall: extracellular vesicles in Gram-positive bacteria, mycobacteria and fungi. Nat. Rev. Microbiol. 13, 620–630 10.1038/nrmicro3480 26324094 PMC4860279

[3] Schwechheimer C. and Kuehn M.J. (2015) Outer-membrane vesicles from Gram-negative bacteria: biogenesis and functions. Nat. Rev. Microbiol. 13, 605–619 10.1038/nrmicro3525 26373371 PMC5308417

[4] Guerrero-Mandujano A., Hernández-Cortez C., Ibarra J.A. and Castro-Escarpulli G. (2017) The outer membrane vesicles: secretion system type zero. Traffic 18, 425–432 10.1111/tra.12488 28421662

[5] O'Donoghue E.J. and Krachler A.M. (2016) Mechanisms of outer membrane vesicle entry into host cells. Cell. Microbiol. 18, 1508–1517 10.1111/cmi.12655 27529760 PMC5091637

[6] Bryant W.A., Stentz R., Le Gall G., Sternberg M.J.E., Carding S.R. and Wilhelm T. (2017) In silico analysis of the small molecule content of outer membrane vesicles produced by *Bacteroides thetaiotaomicron* indicates an extensive metabolic link between microbe and host. Front. Microbiol. 8, 2440 10.3389/fmicb.2017.02440 29276507 PMC5727896

[7] Zakharzhevskaya N.B., Vanyushkina A.A., Altukhov I.A., Shavarda A.L., Butenko I.O., Rakitina D.V. et al. (2017) Outer membrane vesicles secreted by pathogenic and nonpathogenic *Bacteroides fragilis* represent different metabolic activities. Sci. Rep. 7, 5008 10.1038/s41598-017-05264-6 28694488 PMC5503946

[8] Stentz R., Osborne S., Horn N., Li A.W.H., Hautefort I., Bongaerts R. et al. (2014) A bacterial homolog of a eukaryotic inositol phosphate signaling enzyme mediates cross-kingdom dialog in the mammalian gut. Cell Rep. 6, 646–656 10.1016/j.celrep.2014.01.021 24529702 PMC3969271

[9] Lynch J.B. and Alegado R.A. (2017) Spheres of hope, packets of doom: the good and bad of outer membrane vesicles in interspecies and ecological dynamics. J. Bacteriol. 199, e00012-17 10.1128/JB.00012-17 28416709 PMC5512217

[10] Hickey C.A., Kuhn K.A., Donermeyer D.L., Porter N.T., Jin C., Cameron E.A. et al. (2015) Colitogenic *Bacteroides thetaiotaomicron* antigens access host immune cells in a sulfatase-dependent manner via outer membrane vesicles. Cell Host Microbe 17, 672–680 10.1016/j.chom.2015.04.002 25974305 PMC4432250

[11] Ahmadi Badi S., Moshiri A., Fateh A., Rahimi Jamnani F., Sarshar M., Vaziri F. et al. (2017) Microbiota-derived extracellular vesicles as new systemic regulators. Front. Microbiol. 8, 1610 10.3389/fmicb.2017.01610 28883815 PMC5573799

[12] Elhenawy W., Debelyy M.O. and Feldman M.F. (2014) Preferential packing of acidic glycosidases and proteases into *Bacteroides* outer membrane vesicles. mBio 5, e00909-14 10.1128/mBio.00909-14 24618254 PMC3952158

[13] Rakoff-Nahoum S., Coyne M.J. and Comstock L.E. (2014) An ecological network of polysaccharide utilization among human intestinal symbionts. Curr. Biol. 24, 40–49 10.1016/j.cub.2013.10.077 24332541 PMC3924574

[14] Stentz R., Horn N., Cross K., Salt L., Brearley C., Livermore D.M. et al. (2015) Cephalosporinases associated with outer membrane vesicles released by *Bacteroides* spp. protect gut pathogens and commensals against β-lactam antibiotics. J. Antimicrob. Chemother. 70, 701–709 10.1093/jac/dku466 25433011 PMC4319488

[15] Irving A.T., Mimuro H., Kufer T.A., Lo C., Wheeler R., Turner L.J. et al. (2014) The immune receptor NOD1 and kinase RIP2 interact with bacterial peptidoglycan on early endosomes to promote autophagy and inflammatory signaling. Cell Host Microbe 15, 623–635 10.1016/j.chom.2014.04.001 24746552

[16] Shen Y., Giardino Torchia M.L.G., Lawson G.W., Karp C.L., Ashwell J.D. and Mazmanian S.K. (2012) Outer membrane vesicles of a human commensal mediate immune regulation and disease protection. Cell Host Microbe 12, 509–520 10.1016/j.chom.2012.08.004 22999859 PMC3895402

[17] Park J.-Y., Choi J., Lee Y., Lee J.-E., Lee E.-H., Kwon H.-J. et al. (2017) Metagenome analysis of bodily microbiota in a mouse model of Alzheimer disease using bacteria-derived membrane vesicles in blood. Exp. Neurobiol. 26, 369–379 10.5607/en.2017.26.6.369 29302204 PMC5746502

[18] Jang S.C., Kim S.R., Yoon Y.J., Park K.-S., Kim J.H., Lee J. et al. (2015) *In vivo* kinetic biodistribution of nano-sized outer membrane vesicles derived from bacteria. Small 11, 456–461 10.1002/smll.201401803 25196673

[19] Elmi A., Nasher F., Jagatia H., Gundogdu O., Bajaj-Elliott M., Wren B. et al. (2016) *Campylobacter jejuni* outer membrane vesicle-associated proteolytic activity promotes bacterial invasion by mediating cleavage of intestinal epithelial cell E-cadherin and occludin. Cell. Microbiol. 18, 561–572 10.1111/cmi.12534 26451973

[20] Turkina M.V., Olofsson A., Magnusson K.-E., Arnqvist A. and Vikström E. (2015) *Helicobacter pylori* vesicles carrying CagA localize in the vicinity of cell–cell contacts and induce histone H1 binding to ATP in epithelial cells. FEMS Microbiol. Lett. 362, fnv076 10.1093/femsle/fnv076 25956174

[21] Alvarez C.-S., Badia J., Bosch M., Giménez R. and Baldomà L. (2016) Outer membrane vesicles and soluble factors released by probiotic *Escherichia coli* Nissle 1917 and commensal ECOR63 enhance barrier function by regulating expression of tight junction proteins in intestinal epithelial cells. Front. Microbiol. 7, 1981 10.3389/fmicb.2016.01981 28018313 PMC5156689

[22] Günzel D. and Yu A.S.L. (2013) Claudins and the modulation of tight junction permeability. Physiol. Rev. 93, 525–569 10.1152/physrev.00019.2012 23589827 PMC3768107

[23] Soldati T. and Schliwa M. (2006) Powering membrane traffic in endocytosis and recycling. Nat. Rev. Mol. Cell Biol. 7, 897–908 10.1038/nrm2060 17139330

[24] Amano A., Takeuchi H. and Furuta N. (2010) Outer membrane vesicles function as offensive weapons in host–parasite interactions. Microbes Infect. 12, 791–798 10.1016/j.micinf.2010.05.008 20685339

[25] Kaparakis-Liaskos M. and Ferrero R.L. (2015) Immune modulation by bacterial outer membrane vesicles. Nat. Rev. Immunol. 15, 375–387 10.1038/nri3837 25976515

[26] Vercauteren D., Vandenbroucke R.E., Jones A.T., Rejman J., Demeester J., De Smedt S.C. et al. (2010) The use of inhibitors to study endocytic pathways of gene carriers: optimization and pitfalls. Mol. Ther. 18, 561–569 10.1038/mt.2009.281 20010917 PMC2839427

[27] Turner L., Bitto N.J., Steer D.L., Lo C., D'Costa K., Ramm G. et al. (2018) *Helicobacter pylori* outer membrane vesicle size determines their mechanisms of host cell entry and protein content. Front. Immunol. 9, 1466 10.3389/fimmu.2018.01466 30013553 PMC6036113

[28] Izcue A., Coombes J.L. and Powrie F. (2009) Regulatory lymphocytes and intestinal inflammation. Annu. Rev. Immunol. 27, 313–338 10.1146/annurev.immunol.021908.132657 19302043

[29] Rudensky A.Y. (2011) Regulatory T cells and foxp3. Immunol. Rev. 241, 260–268 10.1111/j.1600-065X.2011.01018.x 21488902 PMC3077798

[30] Chu H., Khosravi A., Kusumwardhani I.P., Kwon A.H.K., Vasconcelos C.A., Cunha L.D. et al. (2016) Gene-microbiota interactions contribute to the pathogenesis of inflammatory bowel disease. Science 352, 1116–1120 10.1126/science.aad9948 27230380 PMC4996125

[31] Nieuw Amerongen A.V., Bolscher J.G.M., Bloemena E. and Veerman E.C.J. (1998) Sulfomucins in the human body. Biol. Chem. 379, 1–26 10.1515/bchm.1998.379.1.1 9504711

[32] Derrien M., van Passel M.W.J., van de Bovenkamp J.H.B., Schipper R., de Vos W. and Dekker J. (2010) Mucin-bacterial interactions in the human oral cavity and digestive tract. Gut Microbes 1, 254–268 10.4161/gmic.1.4.12778 21327032 PMC3023607

[33] Nikkari S., McLaughlin I.J., Bi W., Dodge D.E. and Relman D.A. (2001) Does blood of healthy subjects contain bacterial ribosomal DNA? J. Clin. Microbiol. 39, 1956–1959 10.1128/JCM.39.5.1956-1959.2001 11326021 PMC88056

[34] Païssé S., Valle C., Servant F., Courtney M., Burcelin R., Amar J. et al. (2016) Comprehensive description of blood microbiome from healthy donors assessed by 16S targeted metagenomic sequencing. Transfusion 56, 1138–1147 10.1111/trf.13477 26865079

[35] Gosiewski T., Ludwig-Galezowska A.H., Huminska K., Sroka-Oleksiak A., Radkowski P., Salamon D. et al. (2017) Comprehensive detection and identification of bacterial DNA in the blood of patients with sepsis and healthy volunteers using next-generation sequencing method — the observation of DNAemia. Eur. J. Clin. Microbiol. Infect. Dis. 36, 329–336 10.1007/s10096-016-2805-7 27771780 PMC5253159

[36] Lippmann E.S., Al-Ahmad A., Azarin S.M., Palecek S.P. and Shusta E.V. (2015) A retinoic acid-enhanced, multicellular human blood-brain barrier model derived from stem cell sources. Sci. Rep. 4, 4160 10.1038/srep04160 PMC3932448 24561821

[37] Zhao Z., Nelson A.R., Betsholtz C. and Zlokovic B.V. (2015) Establishment and dysfunction of the blood-brain barrier. Cell 163, 1064–1078 10.1016/j.cell.2015.10.067 26590417 PMC4655822

[38] Daneman R. and Rescigno M. (2009) The gut immune barrier and the blood-brain barrier: are they so different? Immunity 31, 722–735 10.1016/j.immuni.2009.09.012 19836264

[39] Branton W.G., Ellestad K.K., Maingat F., Wheatley B.M., Rud E., Warren R.L. et al. (2013) Brain microbial populations in HIV/AIDS: α-proteobacteria predominate independent of host immune status. PLoS ONE 8, e54673 10.1371/journal.pone.0054673 23355888 PMC3552853

[40] Zhan X., Stamova B., Jin L.-W., DeCarli C., Phinney B. and Sharp F.R. (2016) Gram-negative bacterial molecules associate with Alzheimer disease pathology. Neurology 87, 2324–2332 10.1212/WNL.0000000000003391 27784770 PMC5135029

[41] Emery D.C., Shoemark D.K., Batstone T.E., Waterfall C.M., Coghill J.A., Cerajewska T.L. et al. (2017) 16S rRNA next generation sequencing analysis shows bacteria in Alzheimer's post-mortem brain. Front. Aging Neurosci. 9, 195 10.3389/fnagi.2017.00195 28676754 PMC5476743

[42] Padrón F.S., Huergo C.C., Gill V.C., Diaz E.M., Valdespino I.E. and Gotera N.G. (2007) Cuban meningococcal BC vaccine: experiences and contributions from 20 years of application. MEDICC Rev. 9, 16–22 PMID:21487356 10.37757/MR2007V9.N1.6

[43] Oster P., Lennon D., O'Hallahan J., Mulholland K., Reid S. and Martin D. (2005) MeNZB™: a safe and highly immunogenic tailor-made vaccine against the New Zealand *Neisseria meningitidis* serogroup B disease epidemic strain. Vaccine 23, 2191–2196 10.1016/j.vaccine.2005.01.063 15755593

[44] Leca M., Bornet C., Montana M., Curti C. and Vanelle P. (2015) Meningococcal vaccines: current state and future outlook. Pathol. Biol. 63, 144–151 10.1016/j.patbio.2015.04.003 25986879

[45] Davenport V., Groves E., Horton R.E., Hobbs C.G., Guthrie T., Findlow J. et al. (2008) Mucosal immunity in healthy adults after parenteral vaccination with outer-membrane vesicles from *Neisseria meningitidis* serogroup BJ. Infect. Dis. 198, 731–740 10.1086/590669 18636953

[46] Guthrie T., Wong S.Y.C., Liang B., Hyland L., Hou S., Høiby E.A. et al. (2004) Local and systemic antibody responses in mice immunized intranasally with native and detergent-extracted outer membrane vesicles from *Neisseria meningitidis*. Infect. Immun. 72, 2528–2537 10.1128/IAI.72.5.2528-2537.2004 15102760 PMC387915

[47] Bakke H., Setek T.N., Huynh P.N., Haugen I.L., Høiby E.A., Hølst J. et al. (2004) Immunisation schedules for non-replicating nasal vaccines can be made simple by allowing time for development of immunological memory. Vaccine 22, 2278–2284 10.1016/j.vaccine.2003.11.040 15149787

[48] Haneberg B., Dalseg R., Wedege E., Høiby E.A., Haugen I.L., Oftung F. et al. (1998) Intranasal administration of a meningococcal outer membrane vesicle vaccine induces persistent local mucosal antibodies and serum antibodies with strong bactericidal activity in humans. Infect. Immun. 66, 1334–1341 PMID: 9529050 10.1128/iai.66.4.1334-1341.1998 PMC108057

[49] Liu Q., Liu Q., Yi J., Liang K., Hu B., Zhang X. et al. (2016) Outer membrane vesicles from flagellin-deficient *Salmonella enterica* serovar Typhimurium induce cross-reactive immunity and provide cross-protection against heterologous *Salmonella* challenge. Sci. Rep. 6, 34776 10.1038/srep34776 27698383 PMC5048178

[50] Roberts R., Moreno G., Bottero D., Gaillard M.E., Fingermann M., Graieb A. et al. (2008) Outer membrane vesicles as acellular vaccine against pertussis. Vaccine 26, 4639–4646 10.1016/j.vaccine.2008.07.004 18640169

[51] Roy N., Barman S., Ghosh A., Pal A., Chakraborty K., Das S.S. et al. (2010) Immunogenicity and protective efficacy of *Vibrio cholerae* outer membrane vesicles in rabbit model. FEMS Immunol. Med. Microbiol. 60, 18–27 10.1111/j.1574-695X.2010.00692.x 20528929

[52] Schager A.E., Dominguez-Medina C.C., Necchi F., Micoli F., Goh Y.S., Goodall M. et al. (2018) IgG responses to porins and lipopolysaccharide within an outer membrane-based vaccine against nontyphoidal *Salmonella* develop at discordant rates. mBio 9, e02379-17 10.1128/mBio.02379-17 29511082 PMC5844998

[53] Malik B., Goyal A.K., Markandeywar T.S., Rath G., Zakir F. and Vyas S.P. (2012) Microfold-cell targeted surface engineered polymeric nanoparticles for oral immunization. J. Drug Target. 20, 76–84 10.3109/1061186X.2011.611516 21942475

[54] Maerz J.K., Steimle A., Lange A., Bender A., Fehrenbacher B. and Frick J.-S. (2018) Outer membrane vesicles blebbing contributes to *B. vulgatus* MPK-mediated immune response silencing. Gut Microbes 9, 1–12 10.1080/19490976.2017.1344810 28686482 PMC5914909

[55] Kyd J.M. and Cripps A.W. (2008) Functional differences between M cells and enterocytes in sampling luminal antigens. Vaccine 26, 6221–6224 10.1016/j.vaccine.2008.09.061 18852006

[56] Kaufmann S.H.E. (1990) Heat shock proteins and the immune response. Immunol. Today 11, 129–136 10.1016/0167-5699(90)90050-J 2187470

[57] Kim S.-H., Jung D.-I., Yang I.-Y., Kim J., Lee K.-Y., Nochi T. et al. (2011) M cells expressing the complement C5a receptor are efficient targets for mucosal vaccine delivery. Eur. J. Immunol. 41, 3219–3229 10.1002/eji.201141592 21887786

[58] Kimura S., Yamakami-Kimura M., Obata Y., Hase K., Kitamura H., Ohno H. et al. (2015) Visualization of the entire differentiation process of murine M cells: suppression of their maturation in cecal patches. Mucosal. Immunol. 8, 650–660 10.1038/mi.2014.99 25336168

[59] Rangel D.E., Marín-Medina N., Castro J.E., González-Mancera A. and Forero-Shelton M. (2013) Observation of bacterial type I pili extension and contraction under fluid flow. PLoS ONE 8, e65563 10.1371/journal.pone.0065563 23799025 PMC3683016

[60] Xu Q., Shoji M., Shibata S., Naito M., Sato K., Elsliger M.-A. et al. (2016) A distinct type of pilus from the human microbiome. Cell 165, 690–703 10.1016/j.cell.2016.03.016 27062925 PMC4842110

[61] Chen L., Valentine J.L., Huang C.J., Endicott C.E., Moeller T.D., Rasmussen J.A. et al. (2016) Outer membrane vesicles displaying engineered glycotopes elicit protective antibodies. Proc. Natl Acad. Sci. U.S.A. 113, E3609–E3618 10.1073/pnas.1518311113 27274048 PMC4932928

[62] Chen D.J., Osterrieder N., Metzger S.M., Buckles E., Doody A.M., DeLisa M.P. et al. (2010) Delivery of foreign antigens by engineered outer membrane vesicle vaccines. Proc. Natl Acad. Sci. U.S.A. 107, 3099–3104 10.1073/pnas.0805532107 20133740 PMC2840271

